# Endoscopic ultrasound-guided balloon dilation plus platelet-rich plasma for fibrotic ileocecal stricture in Crohn’s disease

**DOI:** 10.1055/a-2853-5951

**Published:** 2026-04-30

**Authors:** Zhengyu Pan, Zeyang Sun, Xiangsu Li, Xin Wang, Yuhai Li, Jinling Tang, Xudong Wu

**Affiliations:** 1 Department of Gastroenterology 612638 Yancheng Clinical Medical College of Jiangsu University, The First People’s Hospital of YanchengYanchengChina; 2 Department of Gastroenterology 612638 The First People’s Hospital of YanchengYanchengChina; 3 Department of Anesthesiology 612638 The First People’s Hospital of YanchengYanchengChina; 4 Department of Digestive Endoscopy 612638 The First People’s Hospital of YanchengYanchengChina; 5 Department of Gastroenterology 612638 The Yancheng Clinical College of Xuzhou Medical University, The First People’s Hospital of YanchengYanchengChina

**Fig. 1:**
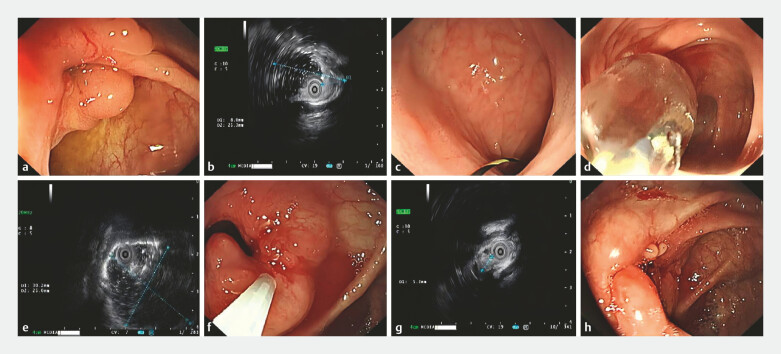
Endoscopic and EUS features of the ileocecal valve stricture across the first and second treatment sessions. **a** An endoscopic view from the first session showing the stenotic ileocecal valve. **b** EUS from the first session demonstrating predominantly hyperechoic submucosal thickening with preservation of deeper mural stratification. **c** Guidewire passage through the stricture during the second session. **d** Through the scope balloon dilation to 18 mm. **e** EUS reassessment after dilation. **f** Circumferential submucosal LP-PRP injection. **g** EUS reassessment after injection. **h** A final endoscopic view confirming luminal patency. EUS, endoscopic ultrasonography; LP-PRP, leukocyte-poor platelet-rich plasma.

A 26-year-old man presented with recurrent postprandial abdominal pain and subobstructive symptoms. Three years earlier, he had been diagnosed with Crohn’s disease (A2L3B2) and had undergone perianal fistula surgery. Despite maintenance treatment with adalimumab and upadacitinib, colonoscopy revealed a deformed, stenotic ileocecal valve with pseudopolyps and superficial erosions, and a standard gastroscope could not traverse the stricture ( Fig. 1 **a** ). After water instillation into the terminal ileum, endoscopic ultrasonography (EUS) demonstrated uneven hyperechoic submucosal thickening with preservation of the muscularis propria and outer wall layers ( Fig. 1 **b** ), supporting staged endoscopic treatment rather than immediate surgery 1 2 3 .

After an initial 15-mm balloon dilation combined with 10 mL leukocyte-poor platelet-rich plasma (LP-PRP), a second session was performed. A 0.035-inch guidewire was advanced across the stricture ( Fig. 1 **c** ), followed by through-the-scope balloon dilation to 18 mm at 5 atm for 60 seconds ( Fig. 1 **d** ). EUS after dilation showed preserved mural stratification without evidence of deep-layer injury ( Fig. 1 **e** ). LP-PRP was then injected circumferentially into the submucosa at five sites (2 mL each) to reduce the restenosis risk ( Fig. 1 **f** , 4 ). Repeat EUS confirmed preserved mural stratification without deep-layer injury and satisfactory injectate distribution ( Fig. 1 **g** ). No perforation or major bleeding occurred. The endoscope subsequently passed through the valve with restoration of luminal patency ( Fig. 1 **h** ). The key therapeutic steps of the second session are shown in Video 1 .

Stepwise 18-mm balloon dilation with EUS reassessment followed by intralesional leukocyte-poor PRP injection for a fibrotic Crohn’s ileocecal valve stricture. EUS, endoscopic ultrasonography; PRP, platelet-rich plasma.Video 1

**Fig. 2:**
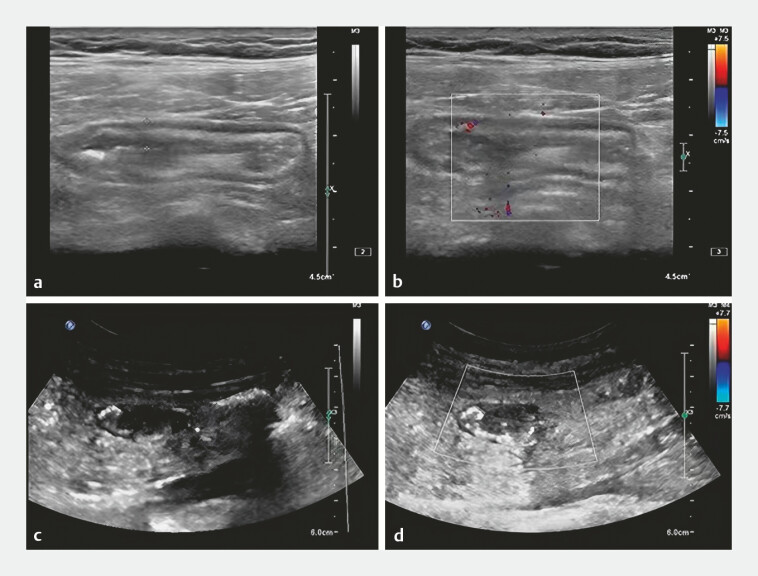
Intestinal ultrasonography follow-up showing improvement in bowel wall thickness from 7.0 mm to 5.6 mm and Limberg grade from 1 to 0.

A third session consisted of LP-PRP alone as maintenance. One month later, symptoms had not recurred and repeat dilation was unnecessary. Intestinal ultrasonography showed improvement in bowel wall thickness from 7.0 mm to 5.6 mm and Limberg grade from 1 to 0 ( Fig. 2 , 5 ).

This case underscores that EUS can help identify fibrosis-dominant Crohn’s strictures, support safe staged dilation, and guide submucosal injection depth. Adjunctive LP-PRP may help maintain patency and defer surgery.

Endoscopy_UCTN_Code_TTT_1AQ_2AH
